# MMP-28 as a regulator of myelination

**DOI:** 10.1186/1471-2202-9-83

**Published:** 2008-09-09

**Authors:** Sean R Werner, Joseph E Dotzlaf, Rosamund C Smith

**Affiliations:** 1 Biotechnology Discovery Research, Lilly Corporate Center, Indianapolis, IN, 46225, USA; 2 Integrative Biology, Eli Lilly and Company, Lilly Research Laboratories, Lilly Corporate Center, Indianapolis, IN, 46225, USA

## Abstract

**Background:**

Matrix metalloproteinase-28 (MMP-28) is a poorly understood member of the matrix metalloproteinase family. Metalloproteinases are important mediators in the development of the nervous system and can contribute to the maturation of the neural micro-environment.

**Results:**

MMP-28 added to myelinating rat dorsal root ganglion (DRG) co-cultures reduces myelination and two antibodies targeted to MMP-28 (pAb180 and pAb183) are capable of binding MMP-28 and inhibiting its activity in a dose-dependent manner. Addition of 30 nM pAb180 or pAb183 to rat DRG cultures resulted in the 2.6 and 4.8 fold enhancement of myelination respectively while addition of MMP-28 to DRG co-cultures resulted in enhanced MAPK, ErbB2 and ErbB3 phosphorylation. MMP-28 protein expression was increased within demyelinated lesions of mouse experimental autoimmune encephalitis (EAE) and human multiple sclerosis lesions compared to surrounding normal tissue.

**Conclusion:**

MMP-28 is upregulated in conditions of demyelination in vivo, induces signaling in vitro consistent with myelination inhibition and, neutralization of MMP-28 activity can enhance myelination in vitro. These results suggest inhibition of MMP-28 may be beneficial under conditions of dysmyelination.

## Background

**Figure 1:**
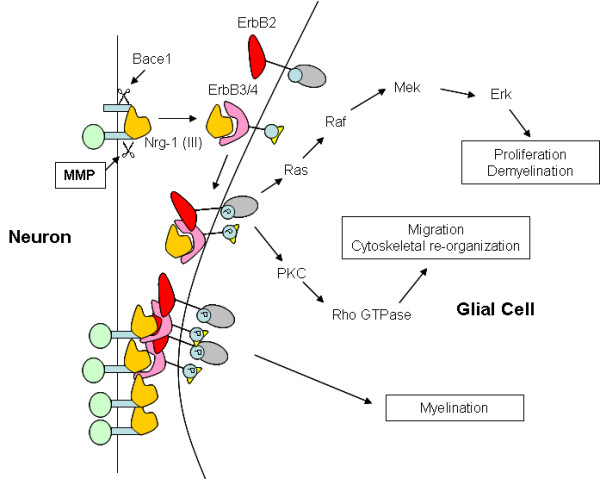
**Myelination signaling**. Neuregulin signaling can lead to a myelinating, proliferative, or migratory response depending on factors such as membrane association or receptor binding. Cleavage of Neuregulin-1 (III) is mediated by Bace1 and MMP proteolysis.

The generation of myelin during development or repair in the peripheral and central nervous systems involves complex signaling between the neuron and the surrounding glial cells [1]. Although the correlation between axon caliber and the elaboration of myelin has been established [2-5], recent studies have started to elucidate the molecular cues that are involved in regulation of myelin formation [6-8]. Axonal Neuregulin-1 (Nrg-1) signaling stimulates either glial proliferation [9] or induces the differentiation of nonmyelinating Schwann cells and oligodendrocytes resulting in myelination depending on localization and amount of Nrg-1 [8]. The explanation of these opposing activities may relate to the downstream signaling pathways activated by Nrg-1. For example, activation of PI3K downstream of Nrg-1/ErbB receptor signaling is required for myelination [10,11]. Alternatively, MAPK activation can also occur following ErbB phosphorylation resulting in inhibition of myelination [11]. The details of the intracellular signaling controlling this balance between proliferation and differentiation are still being elucidated but have been suggested to involve Nrg-1 isoform expression, type I, II, or III [8,12] and proteolysis [8,13,14]. Nrg-1 is cleaved in distinct regions by the β-secretase BACE-1 or by metalloproteinase activity [14]. For example, Nrg-1 type III contains a membrane bound region both C-terminal and N-terminal to the EGF domain. BACE-1 cleaves C-terminal to the EGF domain of Nrg-1 type III allowing access to ErbB 4 receptors while MMP activity cleavage occurs N-terminal to the EGF domain. Cleavage at both sites leads to the generation of a soluble EGF domain [15]. Taveggia et. al. [8] have shown that increased levels of membrane bound Nrg-1 lead to myelination while the proteolytically processed soluble form is proliferative in the PNS (Fig 1). Recently, a role for NRG-1 type III in the promotion of oligodendrocyte mediated myelination has also been shown [16]. MMP activity is known to be important for the proper development of multiple aspects of the neural microenvironment [17]. Data from our laboratory suggests that during development, MMP-28 expression is predominantly neural and peaks in the mouse at embryonic day 14. In addition, protein expression is inversely correlated with the expression of myelin-associated glycoprotein (MAG) during nerve regeneration [18]. Given the temporally regulated pattern of expression of MMP-28 prior to myelination in both developmental and regenerative states, it is likely that MMP-28 plays a functional role in the maturation of nerves. As MMP-28 downregulation precedes myelination and MMP activity is known to regulate molecules related to this process (Neuregulin, Bace-1, ErbB receptors), it is possible that MMP-28 negatively regulates the formation of myelin. This led us to hypothesize that inhibition of MMP-28 activity will result in increased or earlier myelination. Here we show that polyclonal antibodies that recognize two distinct regions of MMP-28 bind recombinant MMP-28 and specifically inhibit its proteolytic activity. In rat primary DRG co-cultures of neurons and glial cells, an *in vitro *model of myelination, these antibodies enhance the expression of axon associated MAG, suggesting a beneficial role of inhibiting MMP-28 during early myelination. Additionally, MMP-28 treatment enhances MAPK phosphorylation, induces rapid phosphorylation of ErbB2 and ErbB3, and reduces phosphorylation of PI3K in myelinating rat DRG co-cultures, changes likely to be inhibitory to the development of myelin. Finally, we demonstrate for the first time that MMP-28 protein levels can be found at increased levels in both mouse experimental autoimmune encephalitis (EAE) spinal cord and in human cerebellar multiple sclerosis lesions. Together, these results suggest that MMP-28 may be a suppressor of myelination and that inhibition of MMP-28 may be beneficial in promotion of myelin repair.

## Results

### MMP-28 added to DRG Co-cultures reduces development of myelin

**Figure 2:**
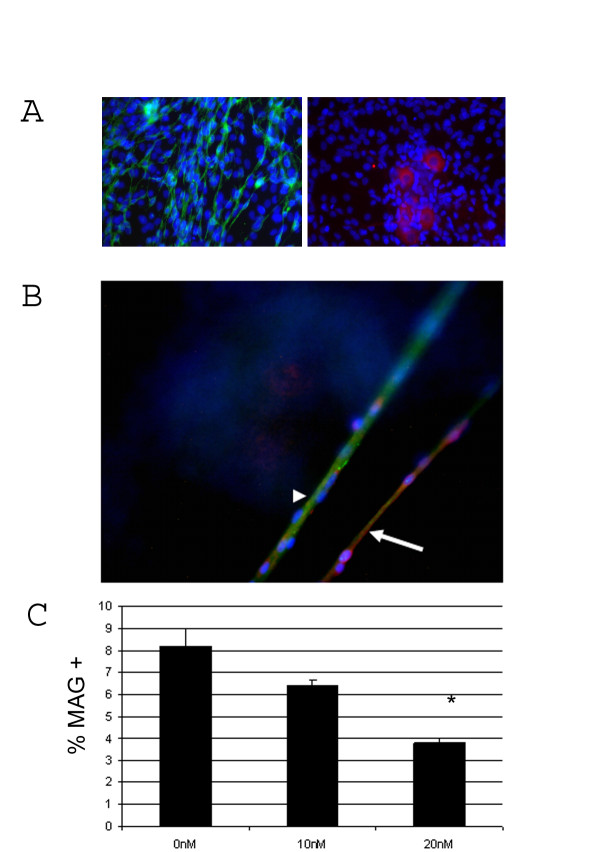
**MMP-28 protein reduces myelination**. A) Identification of cell types present in DRG co-cultures. Green represents S-100 detection, red represents Neurofilament. Nuclei counterstained with DAPI. B) Immunocytochemistry of 6 day myelinating DRG co-cultures. Arrow indicates a representative MAG + axon, arrowhead indicates a representative MAG – axon. Green = MMP-28, Red = MAG. Blue = DAPI. B) Percent of myelinated axons following 6 days of myelination in the presence of 0, 10, or 20 nM MMP-28. *p = 0.006. Data represent the mean of three wells ± Standard deviation.

Previous data from our laboratory suggested that down-regulation of MMP-28 expression in the neuron was permissive for the development of myelin [18] but it is unclear if aberrant MMP-28 expression would effect myelin elaboration. To ascertain if MMP-28 directly inhibits myelination, DRG co-cultures were established and induced to myelinate by the addition of ascorbic acid. Using this system, myelination of axons by Schwann cells can be induced [19]. To verify that our cultures represent PNS cells, S-100, Neurofilament, and Claudin-11 and O4 staining was carried out on 14 day myelinated DRG-derived cells to identify Schwann cells, neurons, and oligodendrocytes respectively (Fig 2A). 64 ± 8% of all cells were identified as Schwann cells by expression of S-100. Less than 1% of the total cells were identified as neurons by Neurofilament staining while O4 and Claudin-11 positive cells were not detected. It remains possible that a small percentage of cells in these cultures are oligodendrocytes however based on morphology and staining, the cultures represent PNS myelination. MMP-28 was included in the media at concentrations of 0, 10 or 20 nM on day 0 and day three following the induction of myelination. MMP-28 treatment during the early stages of myelination resulted in a dose-dependent reduction of axon associated myelin associated glycoprotein (MAG), a marker of myelination, significantly reducing myelin at the 20 nM concentration (Fig 2B–C).

### Polyclonal anti-MMP-28 antibodies bind MMP-28

**Figure 3:**
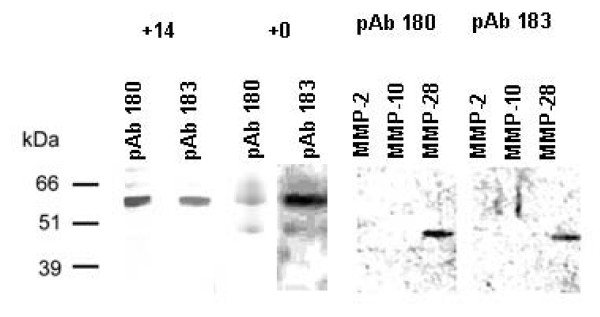
**Polyclonal anti-MMP-28 antibodies recognize MMP-28**. Western blot analysis of 1/10 fraction of total protein isolated from one well of d14 (+14) myelinating rat DRG co-cultures or freshly isolated E17 rat DRGs (+0) or 100 ng of purified MMP-2, MMP-10, or MMP-28. Primary antibody incubation was performed using 50 ng/ml anti-MMP-28 pAb180 and pAb183.

Peptides from regions N- and C-terminal to the active site of human MMP-28 were used to immunize rabbits for the generation of the polyclonal anti-MMP-28 antibodies Ab 180 and pAb 183. Antisera were purified by peptide affinity chromatography followed by Protein G purification. One of the peptides was found to have the closest amino-acid homology to MMP-10 (23%) while no homology was found in the other peptide to any MMPs or other proteins. In binding and activity assays, MMP-10 was used as an MMP containing a related protein sequence and MMP-2 was used as a non-related MMP. To determine antibody specificity, 100 ng of purified human MMP-28, MMP-2, and MMP-10 were loaded on nitrocellulose membranes by SDSPAGE followed by Western blotting. Both antibodies pAb180 and pAb183 were found to recognize the mature form of human MMP-28 (approximately 45 kDa) in samples of purified human MMP-28 protein but did not recognize MMP-2 (72 kDa), or MMP-10 (58 kDa pro-form or 48 kDa mature-form) (Fig 3). The 59 kDa pro-form of MMP-28 in the purified protein sample was detectable upon over exposure (data not shown). The polyclonal antibodies were also found to cross react with rat MMP-28 as determined by Western blot analysis of protein extracts of myelinating rat DRG samples. The 59 kDa pro-form of the protein was detectable in the d14 myelinating rat DRG extracts. The mature form was not detected in these samples, however, 14 day myelinating cultures express low levels of MMP-28 [18] compared to cultures earlier in the myelination program and the cleaved form may not be generated in these cells at this later time point. Bands corresponding to the pro- and mature form of rat MMP-28 were detected by Western blot of freshly isolated E17 DRGs (Fig 3) suggesting that active MMP-28 is present during the early timepoints of the development of myelin in this DRG model of myelination.

### pAb180 and pAb183 inhibit MMP-28 proteolysis

**Figure 4:**
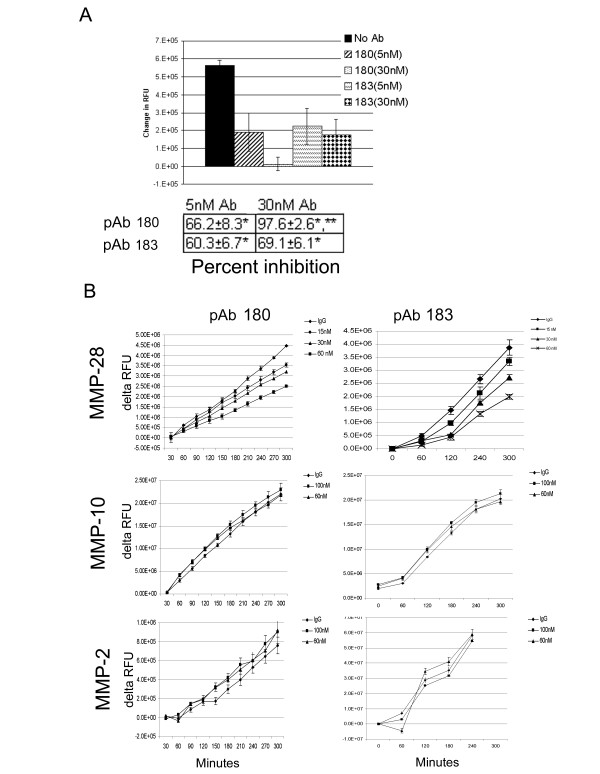
**MMP-28 antibodies specifically inhibit MMP-28**. A) Polyclonal antibodies to MMP-28 inhibit proteolytic activity. 10 nM MMP-28 was pre-incubated for 1 hour at room temperature with 5 or 30 nM anti-MMP-28 pAb180 or pAb183 and subsequently incubated with the Omni-MMP substrate for 24 hours. Data shown as relative fluorescent units or tabulated as % inhibition compared to no antibody controls. Summary of two independent experiments ± SEM (* p ≤ 0.05, ** p ≤ 0.01). B) 100 nM MMP-28, 100 nM MMP-10 or, 10 nM MMP-2 was pre-incubated for one hour at room temperature with pAb180 or pAb183 or IgG control at the indicated concentrations and subsequently incubated with the Omni-MMP substrate. Relative fluorescence was measured at the indicated time points. Summary of two independent experiments ± SEM.

To determine if the anti-MMP28 polyclonal antibodies inhibit MMP-28 activity, 10 nM MMP-28 was pre-incubated with pAb180 or pAb183 at varying concentrations for one hour at 37°C and then assessed for proteolytic activity after 24 hours against an artificial fluorescent pan-MMP substrate. Both antibodies were found to significantly inhibit the activity of MMP-28 at 0.5:1 and 3:1 molar ratio of antibody to MMP28 (Fig 4A). pAb180 inhibited MMP-28 by 66.2%* at 5 nM and 97.6%** at 30 nM while pAb183 inhibited MMP-28 by 60.3%* at 5 nM and 69.1%* at 30 nM (* = p ≤ 0.05, ** = p ≤ 0.01). When measured through 5 hours, enzymatic activity of 100 nM MMP-28 was inhibited in a dose dependent manner by pAb180 or pAb183 while the activity of human MMP-2 (10 nM) or MMP-10 (100 nM) was not inhibited by the addition of 60 or 100 nM pAb180 or pAb183, (Fig 4B). No inhibition was detected in MMP-2 or MMP-10 activity following 24 hours under any of the conditions evaluated (data not shown). These data demonstrate that pAb180 and pAb183 are capable of specifically inhibiting the activity of MMP-28 against a synthetic substrate *in vitro*.

### Anti-MMP28 antibodies enhance myelin formation in vitro

**Figure 5:**
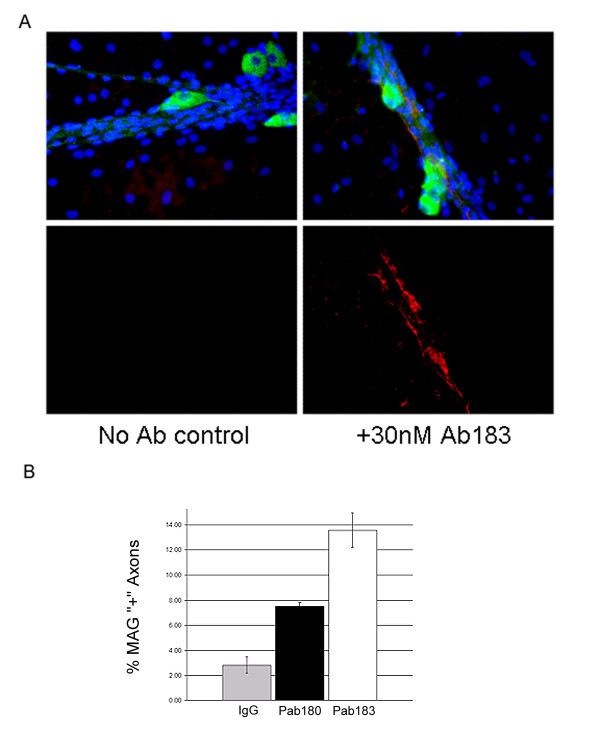
**pAb 180 and pAB 183 increase axon associated MAG**. A) MAG immunofluorescence of myelinating rat DRG co-cultures. Merged or MAG only image of axon bundles of similar size with or without pAb183. Image shows examples of axon bundles determined to be MAG negative (No Ab control) or MAG positive (+30 nM pAb183). Red staining is MAG, Green is MMP-28, Nuclei are stained blue with DAPI. B) Quantification of MAG positive axon bundles following anti-MMP-28 treatment. Data represent mean of 3 independent experiments ± SEM.

Established rat DRG cultures were grown for 6 days under myelinating conditions, an early time point in the development of myelin in this system, at which point 30 nM pAb180 or 30 nM pAb183 was added and the cultures were incubated for 24 hours. MMP-28 is a secreted, membrane associated protein [20], is detectable by immunofluorescence at this time point in DRG co-cultures [18] and is therefore expected to be accessible to anti-MMP-28 antibodies in this system. Assessment of myelination was carried out using a fluorescently labeled antibody to myelin associated glycoprotein (MAG). In previous experiments we have found that a small percentage of axons have associated MAG at this early time point. In all groups, the majority of axons did not have associated MAG; however, antibody treatment resulted in increased MAG expression in some axon bundles (Fig 5A). In untreated cultures, 2.8% ± 0.6% of axon bundles had detectable axon associated MAG staining while in pAb180 or pAb183 treated cultures 7.5% ± 0.3% or 13.6% ± 1.4% of bundles respectively were found to have axon associated MAG expression. This equates to 2.6* and 4.8* fold increases for pAb 180 and pAb183 treated cultures respectively (* = p ≤ 0.05 vs. control) (Fig 5B).

### Alteration of signaling pathways after MMP-28 inhibition

To begin to understand the mechanism responsible for increased myelination seen in DRG cultures with anti-MMP-28 antibodies, we evaluated the ability of MMP-28 to alter signaling pathways that are known to be important in the development of myelin.

**Figure 6:**
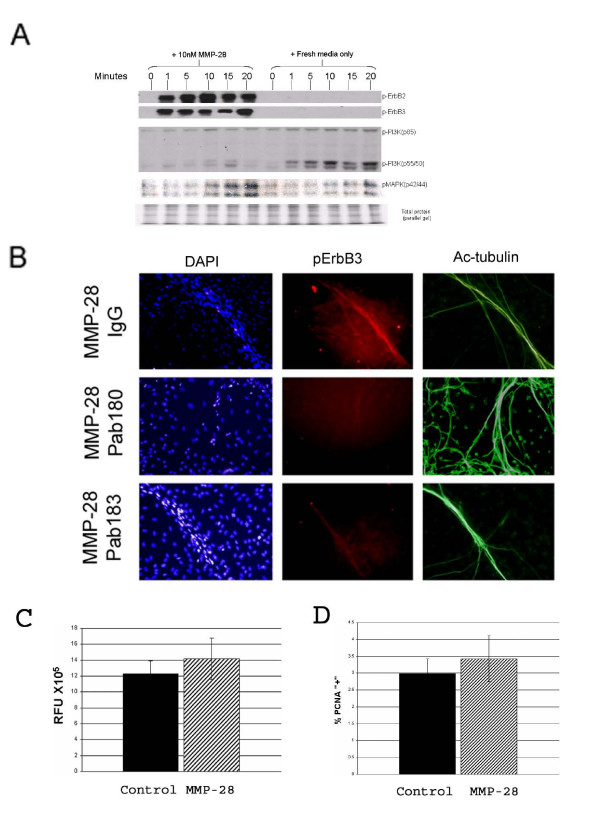
**Signal pathway changes in MMP-28 treated DRG cultures**. A) Western blot analysis of pErbB-2, pErbB3, pPI3K (p55/p85) and phosphoMAPK in rat DRG co-cultures at 0, 1, 5, 10, 15, and 20 minutes following addition of fresh media with or without 10 nM MMP-28. Equal protein loading was determined by Ponceau-S staining of the membrane (not shown) and Coomassie staining of a parallel, equally loaded gel. B) Immunofluorescence of pErbB3 in DRG co-cultures 20 minutes after addition of fresh media containing 10 nM MMP-28 preincubated with 30 nM rabbit IgG, pAb180 or pAb183. Blue:DAPI, Red:pErbB3, Green:Acetylated C,D) Determination of proliferation in DRG co-cultures following MMP-28 treatment using PCNA. C) Relative fluorescence units were counted in 14 day myelinating cultures in control and treated samples. D) Percent of proliferating cells after 2 days in culture was determined by counting PCNA positive nuclei. N = 3, ± SD.

DRG co-cultures were induced to myelinate for 14 days. At this time point endogenous MMP-28 is reduced and a substantial percentage of cells are undergoing myelination and would be expected to have activated myelination related pathways. Cells were then treated with fresh myelination media containing NGF with or with out 10 nM MMP-28. Myelination media contains NGF and is expected to activate signaling pathways including MAPK and PI3K downstream of cognate neurotrophin (Trk or p75) receptors [21] but is not expected to result in phosphorylation of ErbB receptors. The addition of fresh media resulted in a minor increase in phosphorylated MAPK within 10 minutes which was enhanced in the presence of MMP-28 (Fig 6A). In contrast, the phosphorylation of the p55 subunit of PI3K was inhibited in the presence of additional MMP-28 suggesting a resultant decrease in signaling responsible for the development of myelin. Interestingly, both ErbB2 and ErbB3 were rapidly phosphorylated following MMP-28 treatment while the addition of fresh myelination media alone did not result in activation of these receptors. The receptors ErbB2 and ErbB3 are known to be involved in both myelination and proliferation [6,22] however, the presence of MAPK phosphorylation suggests a proliferative rather than myelination related response [23]. As the DRG co-culture system contains a mixed population of cell types, we were interested in determining if the altered signaling occurred in cells involved in the ongoing process of myelination. To identify the cell type with altered signaling following MMP-28 treatment, immunofluorescence was performed on d14 myelinating DRG co-cultures treated with 30 nM IgG alone, or 10 nM MMP-28 with 30 nM IgG, pAb180, or pAb183. Phospho-ErbB3 was not detected in IgG treated control cultures or in cultures treated with MMP-28 in the presence of pAb180 or pAb183 but was detectable along axons in cells of IgG+MMP-28 treated cultures (Fig 6B). While it remains to be shown if the response localized to this area is within the neurons or glial cells, it is clear that the signaling changes do occur in the microenvironment of myelinating cells. The loss of ErbB3 phosphorylation in the presence of inhibitory MMP-28 antibodies suggests that the proteolytic activity of MMP-28 is needed for the observed changes in signaling following treatment. These data indicate that MMP-28 alters the signaling in myelinating DRG cultures, resulting specifically in increased phosphorylation of pathways associated with proliferation rather than myelination in undifferentiated Schwann cells, and that pAb180 and pAb183 inhibit this activity. Phosphorylation of these signaling pathways in myelinating cells within the context of these DRG co-cultures may not be sufficient to elicit a proliferative response, however, to address this possibility DRG co-cultures grown under myelinating conditions for 14 days were treated with 10 nM MMP-28 and analyzed for incorporation of a fluorescently labeled DNA binding agent to detect any increases in DNA content (Fig 6C). No increase in cell number as measured by DNA content was detected 24 hours after MMP-28 treatment. Alternatively, DRG co-cultures prior to myelination induction (2 days in culture) were treated with 10 nM MMP-28 for 24 hours and analyzed for the presence of the proliferation marker proliferating cell nuclear antigen (PCNA) (Fig 6D). No increase in proliferation was detected under these conditions, suggesting that the alteration in signaling due to MMP-28 treatment may not induce proliferation in this system under these conditions.

### Increased MMP-28 protein in demyelinating lesions

**Figure 7:**
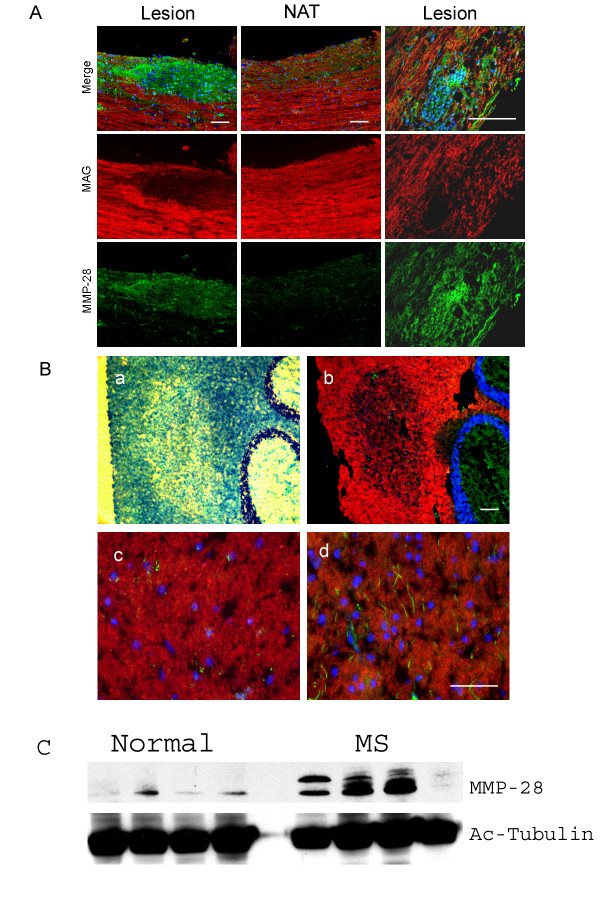
**MMP-28 expression in EAE and MS tissue**. A) Immunofluorescence of mouse EAE spinal cord. Panels represent (from left to right) region with reduced MAG expression (lesion), normal adjacent tissue (NAT) and a separate lesioned area at higher magnification. B) Luxol fast blue and immunofluorescent staining of human cerebellar MS tissue. a: Luxol fast blue staining identifying a lesion containing rarefied myelination. b: Merged MAG (Red), MMP-28 (Green), and DAPI (Blue) staining of MS lesion, c: normal myelinated region (from b), d: region within the lesion demonstrating increased MMP-28 expression (from b). C) Western blot analysis of proteins obtained from cortices of multiple sclerosis patients or normal brains. Four independent normal samples and four independent MS samples are shown.

To determine if MMP-28 is expressed in demyelinating tissues in vivo, MMP-28 expression in two states of demyelination was analyzed. First, demyelination in mice was induced using experimental autoimmune encephalitis (EAE) by immunizing mice with a peptide fragment of myelin oligodendrocyte glycoprotein (MOG). This model of demyelination is a commonly used model for the human demyelinating disease multiple sclerosis (MS)[24]. Expression of MMP-28 and MAG in spinal cord sections from mice with clinical scores of greater than 2 (unilateral hind limb paralysis) showed that MMP-28 was expressed within lesions of reduced MAG expression and at least in part found along axons (Fig 7A). In contrast, MMP-28 expression was found at a lower level in adjacent tissue with continuous MAG staining. This pattern of MMP-28 expression was consistent in the three EAE samples analyzed while MMP-28 was not detected in spinal cord sections from normal, age matched spinal cord sections (data not shown). Second, increased MMP-28 expression appears to occur in brain tissue of human MS patients. Lesions with reduced myelination were identified in cerebellar tissue from a patient with multiple sclerosis by Luxol blue staining (Fig 7B). These regions are in areas of the cerebellum expected to be myelinated and surrounded by normal, myelinated tissue evidenced with more intense Luxol blue staining. MAG staining of serial sections also demonstrated loss of myelin. As expected, in regions identified as normal by Luxol blue staining, MAG protein is strongly expressed indicating normal myelination whereas in the identified lesion, MAG was greatly reduced. Within the boundaries of the identified MS lesions, MMP-28 was detected along individual axons while no such staining was found throughout the normal myelinated regions of the cerebellum. MAG staining was not found to co-localize to these structures. As in the normal mouse spinal cord tissue, no axon associated MMP-28 was detected in normal human cerebellar tissue (data not shown). Additionally, MMP-28, as detected by Western blot in multiple samples, is elevated in cortex taken from MS patients compared to cortex tissue from normal brains (Fig 7C). In the normal samples, only the mature (45 kDa) form of the protein was detected while in the MS samples, increased levels of both the pro- (59 kDa) and the mature form of the protein were detected in some, but not all of the diseased tissues. Further characterization of MMP-28 protein expression in diseased human samples will help to clarify the extent of MMP-28 involvement in multiple sclerosis, however, these results demonstrate that in mouse EAE and human MS tissues, an increase in axon associated MMP-28 can be detected.

## Discussion

We have shown previously that MMP-28 is expressed in the developing nervous system in a temporally regulated manner [18]. This expression is inversely correlated with the expression of MAG in two models of *Xenopus laevis *nerve regeneration and in myelinating rat DRG cultures. These data suggested the possibility that MMP-28 may play a role in the development of myelin during normal development and in neural regeneration. Here we show that exogenously added MMP-28 reduces myelination, that activity *in vitro *can be inhibited by antibodies to MMP-28, and that such inhibition results in enhanced formation of myelin. Specific inhibitors of MMP-28 are not known; therefore, we generated antibodies to two distinct regions of MMP-28 with the goal of developing inhibitory antibodies. The peptides used were unique to MMP-28 and not expected to cross react with other MMPs. In addition, the locations of the epitopes were expected to be near the active site based on the known sequence of MMP-28. Both antibodies were shown to bind and inhibit MMP-28 activity but did not bind or inhibit MMP-2 or MMP-10. MMP-28 is expressed by DRG neurons within the first 14 days after initiation of myelination by ascorbic acid [18]. As MMP-28 expression is downregulated during the period of myelination *in vitro *as well as *in vivo *during nerve regeneration, it is possible that MMP-28 activity is antagonistic to myelination. If so, blocking its proteolytic activity may result in enhancement of the myelination program. Addition of either of the two neutralizing anti-MMP-28 antibodies (pAb 180 or pAb 183) to myelinating DRG cultures resulted in an increase in axon associated MAG staining. This strengthens the hypothesis that neuronal MMP-28 expression after the onset of myelination acts as an inhibitor of the development of myelin. It is not known if MMP-28 acts on the matrix of the neural micro-environment or cleaves cell surface molecules involved in signaling. Illman et al. [20] have shown that MMP-28 is membrane localized, can induce an epithelial-to-mesenchymal transition in lung adenocarcinoma cells and alters signaling mediated through TGF beta. We were therefore interested in the signaling changes that may be mediated by MMP-28 in our cell culture system and chose to evaluate changes in the ErbB activated pathways as signaling downstream of ErbB receptors regulates myelination. Our initial observations suggest that addition of MMP-28 results in rapid phosphorylation of ErbB2 and ErbB3 and enhanced MAPK phosphorylation. Activation of ErbB2 and ErbB3 on glial cells can result in proliferative signals or myelinating signals [22,23] and does not on its own suggest which pathway might be affected. However, ErbB receptor activity in myelinating glial cells is characterized by reduced phosphorylation of MAPK and enhanced PI3K phosphorylation. The enhanced MAPK phosphorylation following MMP-28 treatment in DRG co-cultures coupled with decreased activation of the p55 subunit of PI3K, the active signaling pathway during myelination, are consistent with MMP-28 activity enhancing the non-myelination pathway downstream of the ErbB receptors. It is not yet clear if MMP-28 activity is directly involved in the generation of these intracellular phosphorylation events but there is evidence for MMP-mediated signaling within the nervous system. Of particular interest, both Neuregulin-1 and the ErbB receptors are known to be processed by MMP proteolysis [9,13,25]. As MMP processing of NRG-1 leads to soluble NRG and the myelinating signal is dependent on juxtacrine signaling, it is possible that MMP-28 cleaves NRG-1 in this system and that inhibiting MMP-28 activity results in accumulation of membrane bound NRG-1. In previous studies, we identified increased proteolysis of NCAM and Nogo-A following MMP-28 treated of embryonic rat brains [18]. Alterations in NCAM expression may be involved in the development of myelin [26,27] and Nogo-A, a component of myelin, regulates multiple aspects of glial and neural cell biology [28]. Cleavage of either protein could potentially result in soluble biologically active fragments or loss of function through degradation. Alternatively, the degradation of MAG by MMP-28 is a possibility. Previously, we showed that *in vitro*, at much higher doses than used in these experiments, MAG may be a substrate for MMP-28 [18] and it could be that MMP-28 is degrading MAG expressed in the tissues as it develops until the down regulation of MMP-28. The inverse expression of MMP-28 and MAG suggests that MMP-28 regulation is at least temporally coordinated with the myelination program even if it relates only to the post-transcriptional control of MAG. However, the correlation of MAG staining within myelinated and demyelinated regions of both mouse and human nervous tissue with Luxol Blue staining, which stains the lipid component of myelin, suggests that MAG can be used as marker of myelination even in the presence of varying levels of MMP-28. When taken together with data suggesting that signaling related to the myelination program is altered when exogenous MMP-28 is added, these data support the use of MAG reduction to represent a delay or inhibition of myelination. Further work will be required to determine the essential target of MMP-28 action in vivo.

While the experiments performed *in vitro *demonstrate a role for MMP-28 in regulating PNS myelination, we were curious if a similar role might be involved in the development or maintenance of CNS myelin. We report in this study for the first time that increased MMP-28 expression can be detected within demyelinated lesions of mouse EAE and human MS nervous system tissue. The increase in MMP-28 detected in both cases lends further support to the hypothesis that MMP-28 activity is involved in the regulation of myelin. It can not be determined from these experiments if MMP-28 activity in these tissues is responsible for the demyelination or if it expressed prior to or during any remyelination, however, the timing of the EAE progression in the tissue (21 days after induction) and the increased nuclei (presumably infiltrating immune cells) in both tissues, suggest that these lesions may be actively demyelinating rather than undergoing the process of remyelination. While these experiments represent a small sample size and need to be followed up to determine the specific lesion types that demonstrate this altered expression, the results suggest that MMP-28 may be a relevant target for therapeutic intervention in MS. It is important to note that while there is consistency in MMP-28 expression during development in the CNS and PNS, and between dysmyelination states (PNS renervation, mouse EAE, and MS) the functional implications of altered MMP-28 expression in the CNS may not be the same as in the PNS. Also remaining to be characterized is the role of activation of MMP-28. MMP function is carefully controlled by cleavage of the pro- form to generate the active form of the protein. The sequence of proMMP-28 contains a putative furin recognition sequence but the details of activation for this enzyme *in vivo *are unknown.

Validation of the functional role of MMP-28 in the CNS remains to be carried out. Although the specific mechanism is unknown, MMP-28 appears to play a role in the development of myelin. We suggest that in the nervous system, signaling pathways such as activation of the ErbB-MAPK cascade can be altered in response to MMP-28-induced proteolysis and that continued expression of this protease results in inhibition of robust myelination. If MMP-28 activity plays a similar role in modulating myelination *in vivo*, inhibition of this protease may represent a therapeutic mechanism for enhancing remyelination in demyelinating diseases.

## Conclusion

Although the specific mechanism is unknown, MMP-28 appears to play a role in the development of myelin. We suggest that in the nervous system, signaling pathways such as activation of the ErbB-MAPK cascade can be altered in response to MMP-28-induced proteolysis and that continued expression of this protease results in inhibition of robust myelination. If MMP-28 activity plays a similar role in modulating myelination *in vivo*, inhibition of this protease may represent a therapeutic mechanism for enhancing remyelination in demyelinating diseases.

## Methods

### Generation of polyclonal antibodies

Rabbit polyclonal antibodies were generated to two distinct peptides of human MMP-28 from regions N-terminal or C-terminal to the active site. These epitopes were chosen as they are near the active site of MMP-28 and expected to be accessible to antibodies based on computer modeled predictions of MMP-28 structure. In addition, they are unique to MMP-28 and not expected to bind to other MMPs. Antibodies were affinity-purified from antisera using the corresponding antigen peptides. Antisera were subsequently further purified using protein G spin columns (GE Healthcare) according to the manufacturer's protocol. These purified antibodies, pAb180 and pAb183, were quantified by absorbance at 260 nM and characterized for their ability to bind and modify activity of MMP-28 protein.

### Antibody binding

100 ng of purified recombinant human MMP-28, MMP-2, and MMP-10 were loaded onto a 4–12% bis-tris gel and electrophoresed at 125 V for 90 minutes under non-reducing conditions. For detection of rat MMP-28, 14 day myelinating rat DRG co-cultures grown in 24 well plates were lysed directly in the well with 200 ul of lysis buffer (50 mM, 1 mM EDTA, mM PMSF, 300 mM NaCl, 1 mg/ml BSA, 2% NP-40, with sodium orthovanadate, hydrogen peroxide, and Complete protease inhibitors (Roche) added just before use to final concentrations of 10 μM, 0.01%, and 1× respectively). LDS sample buffer (Invitrogen) was added to a final 1× concentration. One tenth of the total protein prepared from one well was analyzed by Western blot. The proteins were transferred to nitrocellulose membrane at 30 volts for 90 minutes. The membranes with recombinant MMPs or rat protein extract were blocked overnight in 5% non-fat dry milk/TBST at 4°C followed by incubation in 0.5% NFDM/TBST with either pAb180 or pAb183 at 50 ng/ml. Blots were washed 3 times in TBST and incubated in 0.5% NFDM/TBST with an anti-rabbit HRP-conjugated secondary antibody (10 ng/ml) for 1 hour. The blots were then washed 6 times for ten minutes in what in TBST. Chemiluminescent detection was carried out using SuperSignal West-Femto (Pierce, Rockford, IL). Radiographic film was exposed to the blots to detect the presence of MMP-28.

### MMP-28 activity

MMP-28 protein activity was measured using a fluorescently labeled substrate. The fluorescently labeled and quenched peptide, Omni-MMP (Biomol, Plymouth Meeting, PA), a pan-MMP substrate, was dissolved in DMSO to a concentration of 20 mM. MMP protein was diluted in PBS. MMP-28, 10× protease assay buffer (500 mM HEPES pH 7.0, 100 mM CaCl2, 0.5% Brij-35, 100 μM ZnCl2) and substrate were added to a final concentration of 1× assay buffer, 10 μM substrate in 100 μl final volume. Reactions were carried out in black 96 well plates covered with aluminum foil at 37°C. Fluorescence was measured using a Victor3 plate reader (340 nM excitation, 405 nM emission) for 1 second/well.

### DRG cultures

DRG cultures were established as described by Svenningsen et. al. (2003). Embryos were isolated at day 17 of gestation from pregnant Long-Evans rats (Harlan) and DRGs removed. Cells were washed in L15 + 10% FBS 3 times and resuspended in Neuralbasal media (Invitrogen) with 100 ng/ml NGF (BD) and 2% B27 supplement (Invitrogen). Cells were grown on plates coated with Matrigel (1:20 L15) for 4 days after plating at which point fresh media was added with 50 μg/ml ascorbic acid (myelination media) to initiate myelination. Identification of cell type was carried out by immunofluorescent detection of antibodies to S-100 (Dako), Neurofilament (Chemicon), O4 (Millipore), and Claudin-11 (Santa Cruz). Percentage of Schwann cells or neurons was determined by counting nuclei and S-100 or Neurofilament positive cells from three random fields. A minimum of 149 cells were counted per field. Determination of myelination was carried out by immunofluorescent detection of MAG (Chemicon), a marker for the early stages of myelination. Experiments were performed in early myelinating cultures (14 days or less under myelination permissive conditions). To determine the extent of early myelination, cultures were stained by immunocytochemistry for MMP-28 (Cederlane), present in axons (Werner et. al. 2007) to detect axon bundles, DAPI to detect associated Schwann cell nuclei and MAG to detect myelin formation as described previously (Werner et. al. 2007) using fluorescently labeled secondary antibodies. Images were captured of MMP-28, DAPI, and MAG staining from three random fields in each well. Identified axon bundles were determined to be MAG positive or MAG negative. Scoring was performed blinded to treatment group. A minimum of 100 axon bundles per well were counted and myelination represented as MAG positive axon bundles compared to total axon bundle number. Three wells per group were counted. MMP-28 protein was expressed and purified as described previously (Werner et al., 2007) and diluted into myelination media. For the 0 nM MMP-28 treatment, a volume of MMP-28 negative elution fraction equal to the volume used for the 20 nM MMP-28 sample was added to the myelination media.

### Activation of signaling cascades in DRG co-cultures

DRG co-cultures grown in 24 well plates were induced to myelinate for 14 days and then treated with fresh media containing NGF with or without 10 nM MMP-28 (450 ng/ml, 0.5 ml/well). MMP-28 added in the presence of IgG or pAb 180 and pAb 183 was pre-incubated at room temperature for 1 hour prior to addition to cultures. Antibodies added alone to cultures for analysis of myelination were diluted directly in myelination media. Media was aspirated at 0, 1, 5, 10, 15, and 20 minutes after treatment, cells were lysed directly in the wells and subjected to one freeze/thaw cycle at -80°C to disassociate Matrigel. LDS sample buffer (Invitrogen) was added to a final 1× concentration and samples analyzed by Western blot as described earlier. Antibodies were obtained from Cell Signaling Technologies, catalogue numbers as follows: pErbB2 2249S, pErbB3 4784P, pMAPK 4377S, pPI3K 4228P, or LabVision: PCNA Ab-1, PC10. Equal protein loading was confirmed by Coomassie staining of protein in the gel after transfer and Ponceau-S staining of the membrane prior to blocking. For immunoflourescence of phosphoErbB3, cultures were fixed in methanol for 10 minutes followed by three washes in 0.1%Tween 20-PBS (PBST). Nonspecific binding sites were blocked with PBST/5% non-fat dry milk at 37°C for 1 hour followed by incubation with primary antibody (pErbB3, Cell Signaling Technologies No.4784P, acetylated tubulin, Sigma, No. T7451) for 90 minutes at 37°C. Secondary antibodies used were AlexaFluor-488 goat antimouse and Alexa Fluora-555 goat anti-rabbit (Invitrogen). Nuclei are counterstained with DAPI (4',6-diamidino-2-phenylindole, Sigma). To evaluate proliferation in DRG co-cultures, changes in DNA content was measured using the CyQuant NF Cell Proliferation Assay kit (Invitrogen) according to the manufacturers protocol. Briefly, equal number of DRG cells were plated in wells of a 48 well plate and grown under myelination permissive conditions for 14 days at which time, 10 nM MMP-28 or an equal volume of MMP-28 negative column eluate (n = 3 wells per group) was added to the media. After 24 hours, media was removed and 100 μl of 1× dye binding solution was added to the wells and incubated for 60 minutes. Fluorescence was measured on a fluorescent microplate reader (excitation at 485 nm and emission at 535 nm). Proliferation in DRG co-cultures prior to the development of myelin was measured by detection of PCNA by immunofluorescence as described above. Cells were plated at equal number and grown for 2 days at which time 10 MMP-28 or an equal volume of MMP-28 negative column eluate was added for 24 hours. Increases in Schwann cell proliferation have been detected after 24 hours (Lee et al., 1999). Images of three random fields within each well were captured (minimum of 100 cells per field) and total cell number (DAPI stained nuclei) and PCNA positive nuclei were determined.

### Determination of myelination in tissues

EAE was established in 6–8 week old C57BL6 mice by immunization to MOG as follows. MOG35-55(Peptide International, Cat. PMG-3660-PI) and heat inactivated M. Tuberculosis (H37 RA, Difco Laboratories, Cat. 231141) were diluted to a final concentration of 1.5 mg/ml and 2.5 mg/ml respectively in CFA (Complete Freund Adjuvant, Difco Laboratories, Cat. 231131) and emulsified by sonication on ice. Mice were immunized in the right flank by subcutaneous injection of 200 μl of MOG emulsion at day 0 and day 7 followed at t = 0 and t = 48 hours with 200 μl of 2.5 μg/ml Pertussis toxin (List Biological Laboratories, Cat. 181) in PBS. Development of EAE clinical signs was monitored daily according to the following scale: 0 = No clinical EAE symptoms, 0.5 = Distal limp or spastic tail, 1 = Limp tail, 1.5 = Limp tail and hind limb weakness, 2 = Unilateral partial hind limb paralysis, 2.5 = Bilateral partial-hind-limb paralysis, 3.0 = Complete bilateral hind-limb paralysis, 3.5 = Complete hind-limb and unilateral partial-forelimb paralysis, 4.0 = Total paralysis of fore and hind-limbs, 5.0 = Moribund or death. After 21 days, the clinical signs score for treated animals was determined to be at 2 or higher. Immunofluorescence **s**taining was performed on 2 sections from spinal cords of three mice with a score of 2 or greater and three normal control spinal cords. For determination of myelination in human MS, frozen 5 μm cerebellar tissue sections from a human MS patient were obtained (Biomax, Ijamsville, MD). Myelination was detected by Luxol fast blue staining or MAG immunohistochemistry. Paraffin sections were deparaffinized through two changes of xylenes followed by a graded series of methanol and a final wash in PBS while frozen sections were thawed to room temperature before processing. Sections were placed in 0.1% Luxol fast blue solution (0.1 g Luxol fast blue (Acros), 0.5 ml acetic acid, in 95% ethanol to 100 ml) for 16 hours at 56°C. The slides are then removed from Luxol fast blue, washed in 95% ethanol, rinsed in distilled, deionized water (ddH2O) and differentiated in 0.05% lithium carbonate (Acros) for 30 seconds. Following differentiation, slides were then rinsed in ddH2O and examined microscopically to verify differentiation of white matter. The slides were then incubated in 0.1% Cresyl echt violet (American Master Tech Scientific) for 40 seconds to counterstain nuclei and gray matter. Excess Cresyl echt violet was rinsed off the slides with ddH2O. The slides were differentiated in 95% ethanol for 5 minutes followed by sequential dehydration in 100% ethanol and Xylenes. The sections were permanently mounted under coverslips using Permount (Sigma). The tissue was analyzed by light microscopy using an upright microscope. To identify changes in protein expression within MS and EAE lesions, immunohistochemistry was performed using standard techniques with antibodies to MMP-28 (Cedarlane) and MAG (Chemicon). Nuclei were counterstained with DAPI. For Western blot analysis of MMP-28 levels in normal or multiple sclerosis patients, frozen brain tissue samples from multiple sclerosis patients were obtained from the Rocky Mountain Multiple Sclerosis Center and normal brain samples were obtained from in house tissue banks (Eli Lilly). Approximately 5 mg of tissue was cut from the frozen samples and lysed in 500 μl 1× LDS buffer (Invitrogen) containing 1× reducing agent (Invitrogen). 5 μl of each sample was loaded onto a 4–12% Bis-Tris gel and electrophoresed under 100 V for 1 hour. The gel was removed and stained with SimplyBlue (Invitrogen) to verify approximately equivalent protein concentrations. Electrophoresis was repeated and proteins were transferred to nitrocellulose. Western blot detection of MMP-28 was carried out as described above using anti-MMP-28 (Cedarlane). Blots were stripped using Restore western blot stripping buffer (Pierce) according to manufacturers instructions and detection of acetylated tubulin (Sigma) was carried out for normalization of protein levels. All animal use protocols were approved by the Eli Lilly Animal Care and Use Committee.

## Authors' contributions

SRW contributed to the design of experiments, the execution of experiments, the collection and analysis of data and preparation of the manuscript. JED contributed to the design, generation, and analysis of the antibodies and review of the manuscript. RCS contributed to the design and analysis of experiments and contributed to the preparation and review of the manuscript.

## References

[B1] Jessen KR, Mirsky R (2005). The origin and development of glial cells in peripheral nerves. Nat Rev Neurosci.

[B2] Duncan D (1934). The Importance Of Diameter As A Factor In Myelination. Science.

[B3] Friede RL, Miyagishi T (1972). Adjustment of the myelin sheath to changes in axon caliber. Anat Rec.

[B4] Matthews MA (1968). An electron microscopic study of the relationship between axon diameter and the initiation of myelin production in the peripheral nervous system. Anat Rec.

[B5] Voyvodic JT (1989). Target size regulates calibre and myelination of sympathetic axons. Nature.

[B6] Chen S, Velardez MO, Warot X, Yu ZX, Miller SJ, Cros D, Corfas G (2006). Neuregulin 1-erbB signaling is necessary for normal myelination and sensory function. J Neurosci.

[B7] Michailov GV, Sereda MW, Brinkmann BG, Fischer TM, Haug B, Birchmeier C, Role L, Lai C, Schwab MH, Nave KA (2004). Axonal neuregulin-1 regulates myelin sheath thickness. Science.

[B8] Taveggia C, Zanazzi G, Petrylak A, Yano H, Rosenbluth J, Einheber S, Xu X, Esper RM, Loeb JA, Shrager P (2005). Neuregulin-1 type III determines the ensheathment fate of axons. Neuron.

[B9] Garratt AN, Britsch S, Birchmeier C (2000). Neuregulin, a factor with many functions in the life of a schwann cell. Bioessays.

[B10] Maurel P, Salzer JL (2000). Axonal regulation of Schwann cell proliferation and survival and the initial events of myelination requires PI 3-kinase activity. J Neurosci.

[B11] Ogata T, Iijima S, Hoshikawa S, Miura T, Yamamoto S, Oda H, Nakamura K, Tanaka S (2004). Opposing extracellular signal-regulated kinase and Akt pathways control Schwann cell myelination. J Neurosci.

[B12] Lemke G (2006). Neuregulin-1 and myelination. Sci STKE.

[B13] Hu X, Hicks CW, He W, Wong P, Macklin WB, Trapp BD, Yan R (2006). Bace1 modulates myelination in the central and peripheral nervous system. Nat Neurosci.

[B14] Willem M, Garratt AN, Novak B, Citron M, Kaufmann S, Rittger A, DeStrooper B, Saftig P, Birchmeier C, Haass C (2006). Control of peripheral nerve myelination by the beta-secretase BACE1. Science.

[B15] Harrison PJ, Law AJ (2006). Neuregulin 1 and schizophrenia: genetics, gene expression, and neurobiology. Biol Psychiatry.

[B16] Taveggia C, Thaker P, Petrylak A, Caporaso GL, Toews A, Falls DL, Einheber S, Salzer JL (2008). Type III neuregulin-1 promotes oligodendrocyte myelination. Glia.

[B17] Yong VW, Power C, Forsyth P, Edwards DR (2001). Metalloproteinases in biology and pathology of the nervous system. Nat Rev Neurosci.

[B18] Werner SR, Mescher AL, Neff AW, King MW, Chaturvedi S, Duffin KL, Harty MW, Smith RC (2007). Neural MMP-28 expression precedes myelination during development and peripheral nerve repair. Dev Dyn.

[B19] Svenningsen AF, Shan WS, Colman DR, Pedraza L (2003). Rapid method for culturing embryonic neuron-glial cell cocultures. J Neurosci Res.

[B20] Illman SA, Lehti K, Keski-Oja J, Lohi J (2006). Epilysin (MMP-28) induces TGF-beta mediated epithelial to mesenchymal transition in lung carcinoma cells. J Cell Sci.

[B21] Patapoutian A, Reichardt LF (2001). Trk receptors: mediators of neurotrophin action. Curr Opin Neurobiol.

[B22] Guertin AD, Zhang DP, Mak KS, Alberta JA, Kim HA (2005). Microanatomy of axon/glial signaling during Wallerian degeneration. J Neurosci.

[B23] Tapinos N, Ohnishi M, Rambukkana A (2006). ErbB2 receptor tyrosine kinase signaling mediates early demyelination induced by leprosy bacilli. Nat Med.

[B24] Baxter AG (2007). The origin and application of experimental autoimmune encephalomyelitis. Nat Rev Immunol.

[B25] Chandler S, Coates R, Gearing A, Lury J, Wells G, Bone E (1995). Matrix metalloproteinases degrade myelin basic protein. Neurosci Lett.

[B26] Charles P, Reynolds R, Seilhean D, Rougon G, Aigrot MS, Niezgoda A, Zalc B, Lubetzki C (2002). Re-expression of PSA-NCAM by demyelinated axons: an inhibitor of remyelination in multiple sclerosis?. Brain.

[B27] Jakovcevski I, Mo Z, Zecevic N (2007). Down-regulation of the axonal polysialic acid-neural cell adhesion molecule expression coincides with the onset of myelination in the human fetal forebrain. Neuroscience.

[B28] Fontoura P, Steinman L (2006). Nogo in multiple sclerosis: growing roles of a growth inhibitor. J Neurol Sci.

